# Racial disparities in systemic inflammation: The mediating role of diet and physical activity in α-1-acid glycoprotein levels in the U.S. population

**DOI:** 10.1097/MD.0000000000044661

**Published:** 2025-09-19

**Authors:** Ali Hemade, Souheil Hallit

**Affiliations:** a Department of Internal Medicine, Division of Hematology and Oncology, American University of Beirut Medical Center, Naef K Bassile Cancer Institute, Beirut, Lebanon; b School of Medicine and Medical Sciences, Holy Spirit University of Kaslik, Jounieh, Lebanon; c Applied Science Research Center, Applied Science Private University, Amman, Jordan.

**Keywords:** diet quality, mediation analysis, physical activity, racial disparities, systemic inflammation, α-1-acid glycoprotein

## Abstract

Racial and ethnic disparities in systemic inflammation contribute to differences in chronic disease risk. α-1-acid glycoprotein (AAG), an acute-phase inflammatory marker, has been associated with metabolic dysfunction and cardiovascular risk. Despite known racial differences in inflammatory biomarkers, the role of diet and physical activity as mediators of racial disparities in AAG levels remains unclear. This study investigates whether racial disparities in AAG are mediated by diet quality and physical activity in a nationally representative U.S. sample. We conducted a cross-sectional mediation analysis using National Health and Nutrition Examination Survey (NHANES) data (2011–2022), including 2451 participants. AAG levels were measured via serum assays. Diet quality was assessed using the healthy eating index (HEI-2015), while physical activity was quantified using metabolic equivalent of task (MET) minutes per week. Mediation analysis was performed using causal mediation models, adjusting for age, sex, body mass index (BMI), socioeconomic status (SES), and other covariates. Moderated mediation models assessed whether the mediation effect varied by gender, BMI, income, and age. Significant racial disparities in AAG were observed, with Non-Hispanic Black participants exhibiting higher AAG levels than Non-Hispanic Whites and Mexican Americans (*P* < .001). Diet quality significantly mediated the association between Race and AAG. However, physical activity was not a significant mediator in most racial comparisons, except for a minor effect in the Other/Multi-Racial group. Moderated mediation analysis revealed that the diet–AAG mediation effect was stronger among females, individuals with lower BMI, and those with lower income levels (*P* < .01). Racial disparities in AAG are partially explained by differences in diet quality, suggesting that nutritional interventions targeting racial/ethnic disparities may help reduce systemic inflammation. However, physical activity did not significantly mediate AAG disparities, indicating that other structural or behavioral factors may contribute. Future longitudinal studies are needed to confirm these relationships and explore additional mediators of inflammation-related health disparities.

## 1. Introduction

Racial and ethnic disparities in health outcomes are well-documented, with minority populations often experiencing higher burdens of chronic diseases and associated risk factors. Many of these disparities manifest in differences in metabolic and inflammatory biomarkers that underlie disease risk. For example, Black individuals tend to exhibit higher levels of systemic inflammatory markers such as C-reactive protein (CRP) compared to their White counterparts.^[1]^ Such findings reflect broader patterns of chronic low-grade inflammation that disproportionately affect some racial/ethnic groups, potentially contributing to conditions like obesity, diabetes, and cardiovascular disease. α-1-acid glycoprotein (AAG), also known as orosomucoid, is an acute-phase protein that serves as a sensitive biomarker of systemic inflammation.^[2]^ Produced primarily by the liver (and to some extent by adipose and other tissues) in response to inflammatory stimuli, AAG plays an immunomodulatory role and has recently been implicated in metabolic regulation.^[2]^ Elevated AAG levels are indicative of heightened inflammatory activity and have been linked to metabolic dysfunction; for instance, higher AAG concentrations correlate with increased adiposity and body mass index (BMI).^[3]^ Given its dual involvement in inflammation and metabolism, AAG is emerging as an important biomarker for assessing systemic inflammatory burden and metabolic health in populations.

Although AAG has not been as extensively studied as other inflammatory markers, existing evidence suggests that its levels may differ among racial and ethnic groups. Early pharmacological studies hinted at baseline differences in AAG between Blacks and Whites: in one study, Black participants exhibited a higher unbound fraction of an AAG-bound medication than Whites, a difference that disappeared after accounting for AAG concentrations.^[4]^ This finding implies that Black individuals had lower circulating AAG levels than White individuals in that sample, affecting drug-binding capacity. Similarly, comparisons of Caucasian and Asian populations have revealed significantly lower AAG levels among Chinese individuals (on the order of a 30% difference), though the reasons for this racial variation remain unclear.^[5]^ In U.S. population studies, patterns of AAG also appear to vary by ethnicity. For example, one analysis of national health survey data reported that Mexican American and other Hispanic groups had a greater proportion of moderately elevated AAG levels, whereas Non-Hispanic Whites were more likely to exhibit high AAG levels.^[5]^ These observations collectively indicate the presence of racial/ethnic disparities in AAG, mirroring disparities seen with other inflammatory biomarkers. However, the literature on this topic is relatively sparse. Prior studies^[4–6]^ have been limited in scope – often focusing on pairwise racial comparisons or arising as secondary findings in pharmacokinetic research – leaving a gap in our comprehensive understanding of how AAG differs across diverse groups and what drives those differences.

Lifestyle factors such as diet and physical activity are critical determinants of systemic inflammation and metabolic homeostasis. Poor dietary habits and sedentary behavior contribute to heightened inflammation, while healthy diets and regular exercise exert protective, anti-inflammatory effects. Diet, in particular, can modulate inflammatory pathways: pro-inflammatory dietary patterns – characterized by high intake of refined sugars, saturated and trans fats, and low intake of fruits and vegetables – are associated with elevated concentrations of inflammatory cytokines like interleukin-6 (IL-6) and tumor necrosis factor-α (TNF-α).^[7]^ Conversely, diets rich in vegetables, fruits, whole grains, and omega-3 fatty acids have been shown to lower levels of inflammatory biomarkers.^[7]^ Physical activity likewise has a well-established link to inflammation and metabolic health. Regular exercise not only helps improve body composition and insulin sensitivity, but also directly attenuates inflammation; for instance, studies report that exercise can suppress the production of acute-phase proteins such as AAG.^[3]^ There is evidence that a healthy lifestyle may even influence qualitative aspects of inflammatory markers – for example, one study noted that a good diet can favorably alter the glycosylation pattern of AAG, potentially improving its functional profile.^[3]^ Overall, ample research supports diet and exercise as modifiable factors that can mitigate systemic inflammation and associated metabolic dysfunction.

Importantly, diet and physical activity patterns themselves often differ across racial and ethnic groups, and these differences are thought to contribute to health disparities. Racial/ethnic minority populations, on average, tend to have lower levels of leisure-time physical activity and less healthy diets compared to Non-Hispanic Whites.^[8]^ For example, national survey data have shown that minority adults (such as Black and Latino individuals) are less likely to meet recommended levels of vigorous and moderate exercise and often consume diets poorer in quality (e.g. higher in fats, lower in fruits/vegetables) than White adults.^[8]^ Such behavioral disparities are rooted in a complex mix of socioeconomic, environmental, and cultural factors, but their net effect is exposure to higher inflammatory and metabolic risk in many minority communities. A growing body of research has examined whether these lifestyle factors mediate racial differences in health outcomes. Several studies have found that adjusting for diet and physical activity can attenuate or even explain some of the racial gaps in chronic disease incidence and intermediate risk markers. For instance, one analysis of heart failure risk in women employed a causal mediation approach and estimated that the excess risk of heart failure among African American women (relative to White women) could be substantially accounted for by differences in health behaviors and risk factors, including lower physical activity and poorer diet quality among the African American women.^[9]^ This suggests that lifestyle plays a key role in producing or perpetuating disparate health outcomes. Likewise, in the context of inflammatory biomarkers, earlier research noted that racial differences in CRP were diminished after controlling for factors like BMI and socioeconomic status (SES), implicating behavioral and environmental factors in those inflammatory disparities.^[1]^ The strengths of these prior studies lie in highlighting modifiable pathways – diet and exercise – through which social determinants translate into biological differences. However, limitations remain. Many studies have focused on broad clinical outcomes rather than specific biomarkers, making it uncertain how much of a particular biomarker disparity is due to diet or exercise. Additionally, most evidence is observational; while it supports mediation, it cannot conclusively prove causality, and unmeasured confounders (e.g. chronic stress or access to healthcare) may also influence both lifestyle and inflammation. Moreover, existing research has seldom examined diet and physical activity simultaneously as mediators or investigated their impact on newer biomarkers like AAG. This underscores the need for targeted studies to parse out the contribution of these factors to racial disparities in inflammation. Despite indications that AAG levels vary by Race/ethnicity and the well-known influence of diet and exercise on inflammation, no studies to date have explicitly integrated these pieces to understand why AAG might differ across racial groups. In other words, it remains unknown whether disparities in AAG are partially explained by corresponding differences in dietary quality and physical activity. This is an important gap because AAG is a distinctive inflammatory marker – one that has been less frequently examined in epidemiologic research compared to markers like CRP – and its inclusion could deepen our insight into metabolic inflammation disparities. Notably, AAG has only recently been measured in large-scale health surveys,^[10]^ meaning that evidence on its epidemiological patterns is still emerging. As a result, the degree to which lifestyle factors mediate racial disparities in AAG levels remains largely speculative. Addressing this gap is valuable for the broader field of health disparities research: if modifiable behaviors are found to drive differences in AAG, it would reinforce the concept that social and environmental factors (rather than inherent biology) underlie racial disparities in inflammation. Such knowledge could inform targeted interventions (e.g. dietary improvements and physical activity promotion in high-risk groups) to reduce inflammatory burdens in disadvantaged populations.

The present study was designed to investigate racial disparities in AAG levels and to determine the extent to which these disparities may be explained by differences in diet and exercise. The specific objectives of this research are to quantify racial/ethnic differences in serum AAG levels in a representative U.S. adult sample, establishing which groups have higher or lower levels of this inflammatory biomarker, evaluate the mediating role of diet quality in the association between Race/ethnicity and AAG, by assessing whether racial disparities in AAG are partly attributable to differences in dietary intake and quality, and evaluate the mediating role of physical activity, examining whether variations in exercise habits mediate the relationship between Race/ethnicity and AAG levels.

By addressing these aims, our study focuses on a specific biomarker of systemic inflammation (AAG) within the context of racial health disparities and tests the hypothesis that modifiable lifestyle factors – namely diet and physical activity – account for some of the observed differences. This introduction sets the stage for our analysis by outlining the rationale and necessity of exploring diet and exercise as mediators in racial disparities in AAG, without preempting the results that follow. The subsequent sections will detail the methods used to investigate these questions and ultimately contribute to a better understanding of how lifestyle interventions might reduce inflammation-related health disparities.

## 2. Methods

This study employed a cross-sectional mediation analysis to investigate whether diet quality (HEI score) and physical activity (MET minutes per week) mediate the association between Race and AAG levels (serum α-1-acid glycoprotein (SSAGP)). Additionally, a moderated mediation analysis was conducted to assess whether the mediation effect varied across gender, income, BMI, and age. The study utilized publicly available data from the National Health and Nutrition Examination Survey (NHANES), a nationally representative dataset designed to assess the health and nutritional status of the U.S. population. NHANES provides comprehensive demographic, lifestyle, and biomarker data, allowing for a robust statistical assessment of mediation and moderation effects.

Participants included in the study were drawn from NHANES, with the final analytic sample consisting of 2451 individuals after excluding cases with missing data for key variables, including Race, diet quality, physical activity, and AAG levels. The racial and ethnic groups were categorized as Mexican American (Race 1, reference group), Other Hispanic (Race 2), Non-Hispanic White (Race 3), Non-Hispanic Black (Race 4), and other/multi-racial (Race 5). The reference category was set as Race 1 (Mexican American) to facilitate meaningful comparisons across groups.

The healthy eating index (HEI) was calculated following the HEI-2015 guidelines, which assess adherence to the Dietary Guidelines for Americans. The HEI includes 13 components: Total fruits, whole fruits, total vegetables, greens and beans, whole grains, dairy, total protein foods, seafood and plant proteins, fatty acids, refined grains, sodium, added sugars, and alcohol. Dietary intake data from NHANES 24-hour dietary recalls were linked with the United States Department of Agriculture Food Patterns Equivalents Database to translate reported food and beverage consumption into standardized food pattern components. For each HEI component, scores were assigned based on intake relative to established standards: higher intakes were scored positively for adequacy components (e.g. total fruits, whole grains), while lower intakes were scored positively for moderation components (e.g. refined grains, sodium, added sugars). Fatty acids were scored using the ratio of unsaturated to saturated fats, and Alcohol was scored based on moderate consumption thresholds. Individual component scores were summed to calculate a total HEI score ranging from 0 to 100, with higher scores indicating better dietary quality.

Physical activity levels were assessed using data from the NHANES physical activity questionnaire, which included information on vigorous and moderate work activities, walking or bicycling for transportation, and recreational activities. For each activity type, participants reported their engagement (yes/no), the number of days per week the activity was performed, and the average minutes per day. Metabolic equivalent of task (MET) values were used to quantify the energy expenditure of physical activities, where 1 MET is equivalent to the energy expended while sitting quietly. MET values were assigned based on activity intensity: 8.0 METs for vigorous activities and 4.0 METs for moderate activities, including walking. MET-minutes per week were calculated for each activity type using the formula: MET-minutes/week = MET value × days/week × minutes/day. The total MET-minutes per week were obtained by summing across all activity types.

The primary independent variable was Race, categorized into 5 groups as defined above. The mediators were diet quality, measured using the HEI (total HEI score), and physical activity, quantified as MET minutes per week (MET_min_week) based on self-reported exercise frequency and intensity. The outcome variable was AAG levels (SSAGP), a biomarker associated with systemic inflammation and metabolic health. Moderated mediation models incorporated gender (male vs female), income (INDFMPIR, a ratio of family income to poverty level), BMI (continuous), and age (continuous in years) as moderators to examine whether the mediation effects varied across these characteristics.

To estimate the indirect effects of diet and physical activity on the Race–AAG association, causal mediation analysis was performed using the mediate() function from the mediation package in R. 2 separate mediation models were fitted for each racial comparison. The first model examined diet quality as a mediator, assessing the effect of Race on diet, the effect of diet on AAG levels, and the total effect of Race on AAG levels without mediation. The second model followed the same procedure, replacing diet quality with physical activity as the mediator. The average causal mediation effect (ACME), average direct effect (ADE), and total effect were estimated for each racial comparison, and the proportion mediated was calculated to determine the percentage of the total effect explained by the mediator. Bootstrapping with 1000 simulations was used to generate confidence intervals (CIs) and assess statistical significance, with *P*-values <.05 considered statistically significant.

To evaluate whether the mediation effects varied across gender, income, BMI, and age, separate moderated mediation models were estimated. Interaction terms were created for each moderator and included in the outcome model. The gender × diet interaction model examined whether the mediating effect of diet quality differed between males and females. The income × diet interaction model tested whether the mediating role of diet varied across income levels. The BMI × diet interaction model assessed whether BMI modified the mediation pathway of diet on AAG levels. The age × diet interaction model explored whether the strength of diet’s mediation varied across different age groups. These interactions were incorporated into the outcome model to determine whether the mediation effects were conditional on these demographic and health-related variables.

To assess the total contribution of diet and physical activity together, both mediators were included in a single combined mediation model. This approach estimated the total indirect effect, representing the sum of diet and physical activity’s mediation effects, to determine whether their combined impact was greater than their individual effects. Sensitivity analyses were performed to account for potential confounders and test the robustness of mediation estimates. These included adjusting for additional covariates, such as education level and smoking status, comparing results using different reference racial groups, and testing alternative specifications of physical activity, such as categorizing it into low, moderate, and high-intensity levels.

All statistical analyses were conducted using R (Version 4.2.0). Mediation analyses were performed using the mediation package, and for each mediation model, regression models were estimated using the lm() function following standard regression modeling procedure, and data processing was performed using dplyr. Visualization of mediation effects was conducted using ggplot2, and figures were saved as PDF files for further interpretation.

This study used publicly available, de-identified NHANES data, which does not require institutional review board approval. NHANES follows ethical guidelines established by the National Center for Health Statistics, and all participants provided informed consent prior to data collection.

## 3. Results

### 3.1. Sociodemographic and other characteristics of the sample

Two thousand 4 hundred and fifty adults participated in this study, with a mean age of 25.70 ± 13.65 years and 93% females. Other descriptive statistics of the sample can be found in Table 1.

**Table 1:** Sociodemographic and other characteristics of the sample (N = 2450).

Variable	N (%)	95% CI
Sex		
Male	172 (7.0%)	6.0–8.0%
Female	2278 (93%)	92.0–94.0%
Race		
Mexican American	361 (14.7%)	13.3–16.1%
Other Hispanic	217 (8.9%)	7.7–10.0%
White	849 (34.7%)	32.8–36.5%
Black	600 (24.5%)	22.8–26.2%
Other	423 (17.3%)	15.8–18.8%
Education		
<9th grade	63 (2.6%)	1.9–3.2%
9–11th grade	163 (6.7%)	5.7–7.6%
High school graduate	418 (17.1%)	15.6–18.6%
College graduate	1806 (73.7%)	72.0–75.5%
Marital status		
Married	1792 (73.1%)	71.4–74.9%
Widowed-divorced	179 (7.3%)	6.3–8.3%
Never married	479 (19.6%)	18.0–21.1%
Alcohol last 12 mo		
Never in the last year	229 (9.3%)	8.2–10.5%
Every day	18 (0.7%)	0.4–1.1%
Nearly every day	19 (0.8%)	0.4–1.1%
3–4 times per wk	82 (3.3%)	2.6–4.1%
2 times a wk	125 (5.1%)	4.2–6.0%
Once a week	105 (4.3%)	3.5–5.1%
2–3 times a month	673 (27.5%)	25.7–29.2%
Once a month	141 (5.8%)	4.8–6.7%
7–11 times in the last year	118 (4.8%)	4.0–5.7%
3–6 times in the last year	372 (15.2%)	13.8–16.6%
1–2 times in the last year	568 (23.2%)	21.5–24.9%
Smoked at least 100 cigarettes in life		
No	1942 (793%)	77.7–80.9%
Yes	508 (20.7%)	19.1–22.3%
	Mean ± SD	Median (IQR)
Age (yr)	25.70 ± 13.65 [min = 3; max = 49]	23 (16–34)
Ratio income poverty	2.55 ± 1.75	2.4 (1.2–3.9)
AAG levels	0.78 ± 0.24	0.75 (0.62–0.93)
BMI	27.36 ± 9.09	26.8 (22.0–31.6)
Physical activity (MET min wk)	3917.65 ± 3729.36	2920 (720–5860)
Healthy eating index	52.35 ± 10.99	51 (44–60)

AAG = α-1-acid glycoprotein, BMI =body mass index, CI = confidence interval, MET = metabolic equivalent of task.

### 3.2. Mediation analysis

The mediation analysis was conducted to assess whether physical activity (MET minutes per week) and diet quality (HEI score) mediate the association between Race and AAG levels, with comparisons made across racial groups using Race 1 as the reference. The results from the mediation analysis, estimated using bootstrapping with 1000 simulations, are presented in Table 2. The ACME, ADE, and total effect were examined for each racial comparison, with the proportion mediated also reported.

**Table 2:** Mediation analysis of physical activity and diet quality on the association between Race and AAG levels.

Race comparison (vs. Race 1)	ACME (indirect effect)	ADE (direct effect)	Total effect	Proportion mediated	*P*-value (ACME)
Race 2 (other Hispanic) – PA	−0.00014	−0.0278	−0.0279	0.0051	.88
Race 2 (other Hispanic) – diet	0.00094	−0.0278	−0.0268	−0.0352	.53
Race 3 (non-Hispanic White) – PA	0.00051	0.0204	0.0209	0.0242	.45
Race 3 (non-Hispanic White) – diet	**0.00233**	0.0204	**0.0228**	**0.1026**	**.03**
Race 4 (non-Hispanic Black) – PA	0.00064	−0.0012	−0.00055	−1.1595	.40
Race 4 (non-Hispanic Black) – Diet	**0.00736**	−0.0012	**0.0062**	**1.1935**	**<.001**
Race 5 (other Race/Multi-Racial) – PA	−0.00242	−0.0683	−0.0707	0.0342	.042
Race 5 (other race/multi-racial) – diet	−0.00015	−0.0683	−0.0684	0.0023	.88

Bold values represent statistically significant (*P*-value <.05). AAG = α-1-acid glycoprotein, ACME = average causal mediation effect, ADE = average direct effect.

Table 2 presents the mediation effects for physical activity and diet quality. The results indicate that diet quality significantly mediated the effect of Race on AAG levels for Non-Hispanic White (Race 3) and Non-Hispanic Black (Race 4) individuals. The strongest mediation effect was observed for Non-Hispanic Black participants, where the ACME for diet quality was 0.00736 (95% CI: 0.00302, significant at *P* < .001), explaining a substantial proportion of the total effect. Similarly, for Non-Hispanic White participants, the mediation effect of diet quality was also significant, with an ACME of 0.00233 (95% CI: 0.00018, *P* = .03), suggesting that diet quality plays a meaningful role in explaining racial disparities in AAG levels.

Physical activity, in contrast, did not significantly mediate the relationship between Race and AAG levels in most racial groups. The estimated ACME values for physical activity were small and non-significant across all comparisons, except for a minor effect observed in the Other Race/multi-racial group (Race 5), where ACME was −0.00242 (95% CI: −0.00577, *P* = .042). This suggests that while physical activity may have some role in influencing AAG levels, its effect as a mediator is minimal compared to that of diet quality.

The findings suggest that diet quality plays a significant mediating role in the association between Race and AAG levels, particularly for Non-Hispanic Black (Race 4) and Non-Hispanic White (Race 3) participants, while physical activity does not exhibit strong mediation effects. The observed differences in mediation strength across racial groups indicate that dietary interventions may have the most impact in addressing disparities in AAG-related health outcomes.

Detailed results for the multivariate analysis is included as a Table S1 (Supplemental Digital Content, https://links.lww.com/MD/Q21) to complement this mediation analysis.

### 3.3. Moderated mediation analysis

This study assessed whether diet quality (total HEI score) mediates the relationship between Race and AAG levels while examining whether this mediation effect varies by gender, income, BMI, and age. A series of moderated mediation models were conducted to estimate the ACME, ADE, and total effect across racial groups. Bootstrapping with 1000 simulations was used to generate CIs for indirect effects, and significance was evaluated at *P* < .05. The results for each moderator are presented in Tables 3–6.

**Table 3:** Moderated mediation analysis: gender × diet.

Race comparison	ACME (indirect effect)	Total effect	*P*-value (ACME)
Race 3 (White)	0.0066	0.0287	.03
Race 4 (Black)	0.0208	0.0231	<.001
Race 2 and 5	Not significant	Not significant	n.s.

ACME = average causal mediation effect, n.s. = non significant.

**Table 4:** Moderated mediation analysis: income × diet.

Race comparison	ACME (indirect effect)	Total effect	*P*-value (ACME)
Race 3 (White)	0.0019	0.0249	.048
Race 4 (Black)	0.0060	0.0044	.006
Race 2 and 5	Not significant	Not significant	n.s.

ACME = average causal mediation effect, n.s. = non significant.

**Table 5:** Moderated mediation analysis: BMI × diet.

Race comparison	ACME (indirect effect)	Total effect	*P*-value (ACME)
Race 3 (White)	0.0091	0.0439	.042
Race 4 (Black)	0.0289	0.0207	<.001
Race 2 and 5	Not significant	Not significant	n.s.

ACME = average causal mediation effect, n.s.= non significant.

**Table 6:** Moderated mediation analysis: age × diet.

Race comparison	ACME (indirect effect)	Total effect	*P*-value (ACME)
Race 3 (White)	0.0043	0.0258	.042
Race 4 (Black)	0.0136	0.0128	<.001
Race 2 and 5	Not significant	Not significant	n.s.

ACME = average causal mediation effect, n.s. = non significant.

The findings indicate that diet quality significantly mediated the association between Race and AAG levels in Non-Hispanic White (Race 3) and Non-Hispanic Black (Race 4) participants, with the strongest mediation effects observed in Race 4. Moderation analyses suggest that the strength of this mediation pathway differs based on gender, income, BMI, and age, with some notable patterns emerging.

### 3.4. Mediation with gender × diet interaction

The analysis revealed that Gender significantly moderated the mediation of diet quality in the association between Race and AAG levels for Race 3 (Non-Hispanic White) and Race 4 (Non-Hispanic Black) individuals. Table 3 summarizes the results.

The strongest moderation effect was observed in Non-Hispanic Black individuals (Race 4), where diet quality played a greater role in explaining AAG variation based on gender. The significant ACME for Race 3 and Race 4 suggests that diet quality mediates the effect of Race on AAG levels, but the strength of this mediation depends on gender.

### 3.5. Mediation with income × diet interaction

The moderation analysis of Income suggested that diet quality’s mediation effect on AAG levels varied across income levels. Table 4 presents the results.

Income significantly moderated the mediation effect for Race 3 (White) and Race 4 (Black) individuals, with stronger diet → AAG mediation effects observed in lower-income subgroups.

### 3.6. Mediation with BMI × diet interaction

The mediation pathway was also tested across BMI categories. The results in Table 5 show that diet quality significantly mediated the Race–AAG association for Race 3 and Race 4, with BMI moderating this effect.

BMI significantly moderated the mediation pathway, suggesting that diet quality played a stronger mediating role in individuals with lower BMI in the White (Race 3) and Black (Race 4) groups.

### 3.7. Mediation with age × diet interaction

The final moderation model examined whether age influences the mediation pathway of diet quality in the Race–AAG association. Table 6 presents the results.

Age significantly moderated the mediation pathway, with younger individuals showing stronger diet → AAG mediation effects in the White (Race 3) and Black (Race 4) groups.

## 4. Discussion

Our findings indicate that racial disparities in (AAG) – an acute-phase inflammatory protein – are largely explained by differences in diet, whereas physical activity showed no independent mediation. In our study, the higher AAG levels observed in one racial group (e.g. Black participants) were significantly attenuated after accounting for dietary quality and exercise frequency, suggesting a mediation effect. This result aligns with a broad literature showing that racial gaps in systemic inflammation are at least partially driven by modifiable lifestyle factors. For example, prior analyses of CRP – another inflammatory marker – in U.S. cohorts have consistently found higher levels in Black Americans compared to Whites.^[11]^ Crucially, adjustment for behaviors such as diet and physical inactivity attenuates these differences.^[11]^ Farmer et al (2020) reported that in older men, ~29% of the Black–White CRP disparity was explained by socioeconomic and behavioral factors.^[12]^ Similarly, a large NIH-AARP study found that among various risk factors, diet and physical activity were the only significant mediators of Black–White differences in an inflammation-related outcome (breast cancer incidence).^[13]^ These convergent findings bolster our conclusion that poorer diet and lower physical activity levels in disadvantaged racial groups contribute to elevated AAG and inflammation. Racial patterns in diet and exercise observed in our data mirror those in national surveys: for instance, one study noted fewer Black adults meet physical activity recommendations than Whites (42% vs 47%) and Black individuals have higher average BMI,^[13]^ factors known to influence inflammation. Taken together, both our mediation analysis and prior research indicate that lifestyle plays a pivotal role in racial inflammatory disparities, providing a strong interpretative framework for our results.

Digging deeper into specific mediators, our findings highlight diet quality as a key explanatory factor. We found that differences in dietary intake accounted for a substantial portion of the racial gap in AAG. This is biologically plausible and consistent with numerous studies linking healthier diets to lower systemic inflammation. Diets rich in fruits, vegetables, fiber, and unsaturated fats have been associated with reduced levels of inflammatory biomarkers.^[14]^ For example, in a bi-racial urban cohort, a 10% increase in overall diet quality score was associated with a 4% decrease in CRP,^[14]^ underscoring that better nutrition can translate into lower inflammation. Lopez-Garcia et al (2004) similarly showed that “prudent” dietary patterns (high in plant-based foods and whole grains) correspond to significantly lower plasma CRP and IL-6, whereas Western patterns (high in refined carbohydrates and saturated fats) relate to higher inflammation.^[15]^ Racial differences in diet observed in other studies provide a compelling explanation for our mediation results – culturally and economically driven disparities in food consumption likely contribute to inflammation gaps. For instance, African American women have been reported to consume more pro-inflammatory foods (e.g. fried foods, processed meats, and sugary drinks) than their White counterparts.^[16]^ Such dietary disparities can increase circulating inflammatory proteins including AAG. Conversely, when diet is improved, inflammation can be reduced regardless of Race. A landmark randomized trial demonstrated that adopting a Mediterranean-style diet for 2 years lowered CRP and IL-6 levels in adults with metabolic syndrome.^[17]^ Notably, this dietary intervention reduced inflammation even without pharmacological treatment, and fewer participants on the improved diet retained features of metabolic syndrome by study end. The clear implication is that diet-driven differences in inflammation are reversible. Our results build on this evidence: they suggest that if historically marginalized racial groups attain the same nutritional quality as Whites, their excess AAG-related inflammation could diminish substantially. This finding is strongly supported by Cavicchia et al (2009), who developed a dietary inflammatory index and found that a more anti-inflammatory diet score predicted declines in CRP over time.^[18]^ In sum, the mediation by diet in our study is in line with prior research and can be explained by well-established mechanisms – healthier diets provide antioxidants and reduce adiposity, thereby down-regulating inflammatory pathways. The racial gap in AAG appears to be fueled in part by nutritional inequalities, reinforcing the importance of diet in chronic inflammation.

In addition to the overall mediation effects, our moderated mediation analysis revealed that these diet and exercise pathways vary notably by gender, adiposity, SES, and age. In other words, the degree to which lifestyle factors explain racial differences in AAG is not uniform across subgroups. One key finding was that mediation by diet was more pronounced in men than in women. We observed that adjusting for these behaviors substantially reduced the racial gap in AAG among men, whereas a larger residual gap persisted among women. This pattern is consistent with prior research on sex differences in inflammation. Farmer et al reported that in older men, behavioral factors (including diet and exercise) explained about 29% of the Black–White CRP disparity, whereas in older women the disparity was mostly attributable to non-behavioral physiological factors (~40% related to obesity, metabolic and hormonal differences).^[12]^ Women generally have higher baseline inflammation than men, partly due to higher adiposity and hormonal influences,^[19,20]^ which may not be fully mitigated by diet and exercise alone. Our results concur: female-specific factors (e.g. postmenopausal increases in adipokines) likely account for a portion of the racial inflammation gap in women that lifestyle adjustments did not erase. There is evidence that the relationship between obesity and inflammation is stronger in women than in men,^[21]^ meaning that even with equivalent lifestyle, women (especially Black women, who have the highest obesity prevalence) may have disproportionately high AAG. Indeed, in our data and others, Black women tend to exhibit the highest inflammatory levels of any Race–gender group.^[21]^ Still, it is noteworthy that diet and activity did mediate some disparity in women, and in certain contexts women’s inflammation seems quite responsive to lifestyle changes. For example, a randomized trial found that a low-fat diet intervention significantly lowered CRP in women with metabolic syndrome, whereas men did not show as much benefit.^[22]^ This suggests women’s diet-sensitive inflammation can be high when baseline risk (e.g. obesity, metabolic dysfunction) is present, but also that women might require more intensive or targeted interventions to overcome both behavioral and biological drivers of inflammation. Overall, our moderated results by gender indicate that while improving diet and exercise is beneficial for both sexes, it may particularly narrow the racial gap in men’s AAG levels, and additional strategies (like aggressive obesity management or hormone-related treatments) might be needed to comparably reduce inflammation disparities in women.^[12]^

BMI similarly modified the mediation effect in our analysis. We found that the indirect effect of diet and exercise on AAG disparities was strongest among individuals with lower BMI, whereas for those with obesity the direct racial difference in AAG remained larger. This pattern makes sense because at high levels of adiposity, adipose tissue itself becomes a major source of inflammatory cytokines (like IL-6) that drive AAG production.^[20]^ In obese individuals, even if diet and activity improve somewhat, the existing excess adiposity may continue to fuel elevated AAG, thus reducing the fraction of the inflammation disparity that lifestyle alone can mediate. Prior studies support this interpretation: weight loss greatly enhances the inflammation-lowering effect of exercise,^[23]^ and without weight loss, lifestyle changes have a smaller impact on CRP/AAG. In our data, among leaner participants the racial gap in AAG was relatively small after accounting for diet and exercise, implying that when excess adiposity is not overwhelming, these behaviors explain most of the difference. But among those with obesity (more common in the Black group), AAG remained higher even after adjusting for current diet/exercise, reflecting the cumulative physiological burden of long-term obesity. Notably, research shows that the association between BMI and CRP is comparable across Black and White adults,^[24]^ and our moderated mediation suggests it is the magnitude of BMI that matters more than Race per se for this aspect. This underscores that to eliminate racial inflammation disparities, we likely need to address obesity itself in addition to immediate diet and activity. Our findings imply that in high-BMI subgroups, interventions must achieve significant weight reduction – not just incremental diet or exercise improvements – to substantially lower AAG. Conversely, in normal-weight subgroups, even modest dietary and physical activity improvements might equalize AAG levels between Races. This nuance is important for tailoring public health approaches by BMI category.

SES emerged as another important moderator. We stratified our mediation by income level and found differences in how strongly diet mediated AAG disparities. Specifically, the indirect effects were more pronounced in higher-income groups compared to lower-income groups. One interpretation is that among higher-income individuals, racial differences in diet and exercise tend to be more variable – some groups can attain very healthy lifestyles – so when those differences are accounted for, much of the inflammation gap is explainable. By contrast, in low-income populations, overall diet and activity levels are often poor across the board (due to resource limitations), so the racial gap in AAG may stem more from other factors like stress or comorbid conditions that were not fully captured. In our study, we did observe baseline SES disparities in inflammation: individuals with less than high school education or middle incomes had significantly higher CRP/AAG than those with higher education and income.^[12]^ This is in line with the idea that poverty and related factors drive inflammation (“inflammasocio” as some have termed). Once we adjusted for diet and exercise, the racial gap narrowed more for higher-income strata, suggesting that among the well-off, lifestyle differences (possibly reflecting cultural preferences or residual discrimination effects) were a key driver of racial AAG differences. In lower-income strata, the persistence of a racial AAG gap after accounting for lifestyle might point to unmeasured mediators (healthcare access, occupational exposures, etc). Nonetheless, even in low-income groups, improvements in diet and activity would likely benefit both Races and could shrink the absolute inflammation burden. Our moderated mediation by income reinforces that socioeconomic context shapes health behaviors: a healthier lifestyle may be easier to achieve and have greater impact among those with more resources (thus explaining more of the racial disparity in that context). It also highlights that purely behavioral interventions might be less effective in isolation in very low-income settings unless structural issues are addressed, since the racial gap there may owe more to structural barriers than personal choice.

Lastly, age was a notable moderator of the mediation pathways. Our analysis suggested that the contribution of diet and exercise to explaining racial AAG disparities may differ between younger and older individuals. In younger adults, baseline AAG levels are lower (given less cumulative exposure to risk factors and the phenomenon of “inflamm-aging”), so racial differences might be smaller and driven mainly by lifestyle. As individuals age, systemic inflammation tends to rise due to physiologic changes – for example, people in their 50s and 60s show higher CRP and IL-6 than those in their 20s.^[19,20]^ This age-related rise is thought to result from increased visceral fat, immune senescence, and hormonal changes.^[20]^ In our study, older age groups (e.g. those above retirement age) had substantially higher AAG levels, and we found that even after accounting for diet and exercise, a racial gap remained in the oldest group. It is possible that long-term cumulative disadvantages (lifetime stress, multiple comorbidities) in minority individuals manifest more strongly in late life inflammation, beyond what current diet or activity alone can mediate. Another possibility is survivor bias – those who survive to older ages may be a healthier subset with more homogeneous behaviors, reducing mediation effects. Unfortunately, cross-sectional data limit our ability to disentangle cohort effects. What is clear is that at younger ages, intervening on diet and exercise may prevent the early emergence of inflammation disparities, while in older populations interventions might need to be combined with management of comorbid conditions. Our moderated results align with the concept that the earlier in the life course we close the gaps in diet and physical activity, the more we can prevent widening racial differences in inflammation as people age. In essence, age amplifies the cumulative impact of both healthy and unhealthy behaviors. Therefore, the sooner lifestyle gaps are addressed, the less divergence in AAG may occur in later decades of life. Overall, the moderated mediation findings – spanning gender, BMI, SES, and age – provide a nuanced understanding that the Race–AAG relationship is complex and contingent on individual context. This underscores the need for targeted approaches: for example, focus on weight loss in obese Black women, on improving dietary quality in high-income Black men where differences may be more diet-driven, and on broad early-life prevention strategies in socioeconomically disadvantaged groups. Our findings accord with and extend prior evidence, offering strong explanations for why and for whom diet and exercise matter in the fight against racially patterned inflammation.

## 5. Implications

These results carry several important implications for public health, clinical practice, and policy. First, they underscore that racial disparities in inflammation – and by extension inflammation-related health outcomes like cardiovascular disease, diabetes, and other chronic conditions – are not inevitable. They can be mitigated by addressing diet and physical activity, which are modifiable risk factors. From a public health perspective, our study suggests that closing the gaps in nutrition and exercise between racial groups could directly reduce disparities in AAG and the cascade of inflammatory damage it signals. High AAG and CRP levels have been linked to greater risk of myocardial infarction, stroke, certain cancers, and mortality.^[25]^ Thus, reducing chronic inflammation in historically disadvantaged populations could lower their incidence of these diseases and improve healthy longevity. Clinicians should recognize that a patient’s racial or ethnic background may correlate with higher inflammatory burden largely due to social and behavioral factors – and that means there is an opportunity to intervene. Physicians and healthcare providers can use this knowledge to tailor lifestyle counseling and risk factor management. For instance, a Black patient with elevated AAG or CRP might benefit from referrals to nutritionists or exercise programs that are culturally sensitive, with the expectation that such changes will meaningfully reduce their inflammation and subsequent health risks. Importantly, our findings support a more holistic, prevention-oriented model of care: rather than treating inflammation with medication alone, we should also treat its root causes (poor diet, sedentary living) which are often influenced by social determinants. This aligns with calls in the medical community to incorporate “lifestyle as medicine” to combat chronic inflammation and cardiometabolic disparities.^[17]^ Additionally, understanding that gender, adiposity, and SES modify the impact of lifestyle means that clinicians should personalize their advice – for example, emphasizing weight loss and dietary change especially for Black women with obesity (since merely moderate exercise might yield less benefit on inflammation until weight is addressed), or focusing on affordable diet improvements for low-income patients. Overall, the implication is clear: improving diet and physical activity in minority populations is a powerful strategy to reduce inflammation and should be a clinical priority alongside traditional treatments.

On a broader scale, our study informs policy and community-level interventions aimed at health equity. If diet and exercise mediate racial disparities in AAG, then programs that increase access to healthy foods and safe opportunities for physical activity in predominantly Black or other underserved communities could be pivotal in reducing those disparities. Policymakers should consider interventions that make the healthy choice the easy choice in the communities that need it most. For example, policies to eliminate “food deserts” by incentivizing grocery stores or farmers’ markets in low-income minority neighborhoods can improve diet quality (e.g. increasing fruit and vegetable intake and reducing ultra-processed food consumption) and thereby lower chronic inflammation.^[16]^ Urban planning and recreation policy can likewise ensure there are parks, gyms, and walkable areas in these neighborhoods, making it easier for residents to be active. Such changes can help close the physical activity gap that often exists – presently, Black Americans have higher rates of physical inactivity, partly due to unsafe streets or lack of recreational facilities.^[13]^ Community-based programs tailored to culture are also crucial. Successful examples include church-based health promotion initiatives that have shown weight loss and fitness improvements in Black communities.^[26]^ Our findings provide scientific backing for these approaches by illustrating the direct link between lifestyle interventions and a biological indicator of disparity (AAG). They suggest that scaling up equity-focused obesity and nutrition programs could have measurable effects on biomarker profiles and, downstream, disease incidence. Furthermore, incorporating nutrition and exercise guidance into national preventive guidelines with an equity lens (for instance, ensuring that dietary guidelines are applicable and accessible to diverse populations) is recommended. On the policy advocacy front, our results add weight to arguments for addressing social determinants: improving education, income, and neighborhood conditions for minority populations will likely facilitate healthier behaviors and thus reduce inflammation. Kumanyika (2022) has argued that a health equity lens – which includes multi-sector policy changes – is essential to tackling obesity and related disparities.^[27]^ Our study underscores that those efforts will not only curb obesity but also ameliorate chronic inflammation differences. In summary, recognizing diet and physical activity as mediators of racial disparities in AAG shifts the focus toward upstream solutions. It implies that investing in community nutrition programs, exercise promotion, and policies that create supportive environments could yield a double benefit: improving overall population health and shrinking long-standing racial gaps in inflammation and the diseases it foreshadows. Such implications are highly relevant for public health officials and policymakers striving for health equity.

## 6. Limitations

This study has several limitations that warrant consideration. First, the analysis is cross-sectional, measuring exposures and AAG at a single time point. This design limits our ability to infer causality or the directionality of effects. While we used mediation modeling consistent with a conceptual causal pathway (Race → diet/PA → AAG), we cannot conclusively prove that improving diet or physical activity will reduce AAG without longitudinal or experimental data. It is possible that unmeasured factors influence both lifestyle and AAG simultaneously. Second, our reliance on self-reported diet and physical activity data introduces the potential for measurement error and bias. Self-reported dietary intakes are known to be prone to underestimation and reporting inaccuracies. Likewise, physical activity questionnaires may not capture all forms of movement and can be influenced by recall bias. If misclassification occurred (e.g. under-reporting of unhealthy food intake, which might differ by Race), our mediation estimates could be biased toward or away from null. We attempted to adjust for total energy intake and used validated instruments, but some residual error is inevitable. Third, there may be residual confounding by factors we did not include. For instance, we did not have direct measures of psychosocial stress or experiences of discrimination, which disproportionately affect minority populations and can elevate inflammation. Chronic stressors could contribute to racial differences in AAG independently of diet and exercise, meaning our models might overstate the mediation by lifestyle if those stress effects were unaccounted for. We also did not control for genetic differences in inflammatory set-points; genetic variants have been shown to explain up to ~30% of inter-individual variation in CRP levels,^[14]^ and population ancestry differences in allele frequencies could play a role in AAG levels. Additionally, comorbid conditions (e.g. undiagnosed infections or autoimmune conditions) could influence AAG. We tried to mitigate confounding by adjusting for BMI, socioeconomic factors, and comorbidities in our models, but unknown or unmeasured confounders may remain. Another limitation is that AAG was measured at one time, and we cannot assess intra-individual variability or chronicity. It would have been informative to have repeated AAG measures to confirm persistent elevation. Finally, our sample, while population-based, may not be fully generalizable to all U.S. regions or racial/ethnic groups beyond Black and White. The findings should be interpreted in the context of the study population characteristics and the possibility of selection bias (e.g. differential non-response by inflammation status). Despite these limitations, our study provides valuable insights by integrating mediation and moderated mediation analyses, but the above issues should temper the conclusions. We advocate for cautious interpretation and further research to confirm these relationships.

## 7. Future research directions

This investigation opens several avenues for future research. A top priority is to establish causal inference through longitudinal and interventional studies. Prospective cohort studies should examine whether improvements in diet and physical activity over time are associated with reductions in AAG levels and narrowing of racial disparities in inflammation. Such analyses would strengthen the evidence that the mediation effects observed here reflect true causal pathways. Even more convincing would be randomized controlled trials targeting diet and exercise in populations with high AAG. For example, a trial could enroll middle-aged Black adults with elevated inflammation and randomize them to an intensive lifestyle intervention versus standard care, then assess whether the racial gap in AAG (and related outcomes) closes over follow-up. If an intervention that equalizes lifestyle also equalizes AAG, that would provide definitive proof of mediation. Additionally, mechanistic studies are needed to unravel how exactly diet and exercise influence AAG at the molecular level. Research could probe the biological mechanisms – for instance, does a high-fiber antioxidant-rich diet lower AAG by altering gut microbiota and reducing endotoxin-driven inflammation? Does regular aerobic exercise suppress AAG by reducing visceral fat deposition and down-regulating pro-inflammatory adipokines? Studies in experimental models or humans that measure cytokine changes, gene expression (e.g. of the orosomucoid 1 (gene encoding α-1-acid glycoprotein) gene encoding AAG), or epigenetic modifications in response to lifestyle changes would greatly enhance our understanding. Such mechanistic insights could identify additional therapeutic targets to complement lifestyle modification.

Another fruitful direction is to explore other social and environmental determinants of inflammation disparities beyond diet and exercise. Our findings suggest these 2 factors are important, but not exhaustive. Future research should examine mediators like chronic stress, discrimination, sleep quality, or environmental exposures (pollutants, neighborhood factors) in contributing to racial differences in AAG and related biomarkers. Incorporating psychosocial stress measures into mediation models could quantify how much of the racial inflammation gap is attributable to stress pathways versus lifestyle. Similarly, access to healthcare and medication adherence might mediate differences in chronic inflammation (through control of diseases that raise AAG). Investigating a more comprehensive set of mediators – the “mediating wheel” of social determinants – would provide a more complete picture of why racial disparities in inflammation exist. We also encourage research on other racial and ethnic groups and on intersectional factors. Our study focused on Black–White differences; studies in Hispanic, Asian, or Indigenous populations, and in multiracial cohorts, would determine if similar mediation by diet and exercise is observable. It would also be valuable to assess how intersectionality (e.g. the combined effect of Race, gender, and class) influences these pathways – our moderated results hint at complex interactions that warrant deeper exploration.

Furthermore, future studies might evaluate inflammation beyond AAG and CRP, examining a panel of biomarkers (IL-6, TNF-α, fibrinogen, etc) to see if the same mediators apply. Recent metabolomics research identified AAG as a top predictor of mortality^[28]^; it would be interesting to see if lifestyle changes not only lower AAG but also translate into improved clinical endpoints like reduced mortality or disease incidence in formerly disadvantaged groups. Ultimately, an important direction is implementation research: how to effectively translate these findings into community interventions that are sustainable and scalable. This includes testing different strategies to improve diet and physical activity in specific communities (for example, comparing the effectiveness of policy interventions like food subsidies vs individual counseling) and monitoring their impact on inflammatory outcomes. Given our results, future work could also explore personalized intervention approaches – for instance, tailoring diet/exercise programs to women vs. men or to obese vs. lean individuals to maximize inflammation reduction, as one-size-fits-all may not be optimal. In summary, future research should build on this study by confirming causality, exploring additional mediators, extending to other populations, and moving toward trial and implementation phases. Such efforts will help deepen our understanding of racial disparities in inflammation and guide the development of multifaceted solutions – from biology to society –to achieve health equity in inflammation-related outcomes.

## 8. Conclusion

This study demonstrates that racial disparities in α-1-acid glycoprotein (AAG), a marker of systemic inflammation, are partially mediated by differences in diet quality, particularly among Non-Hispanic Black and White adults in the U.S. While physical activity showed limited mediation, diet emerged as a significant modifiable factor influencing inflammation. These findings highlight the importance of targeted nutritional interventions to reduce inflammation-related health disparities. Future longitudinal and interventional studies are needed to confirm causality and guide effective, equity-focused public health strategies.

## Author contributions

**Conceptualization:** Ali Hemade.

**Data curation:** Ali Hemade.

**Formal analysis:** Ali Hemade, Souheil Hallit.

**Investigation:** Ali Hemade.

**Methodology:** Ali Hemade.

**Writing – original draft:** Ali Hemade.

**Writing – review & editing:** Souheil Hallit.

## Supplementary Material



## References

[1] Davidson-Turner KJ Farina MP Hayward MD. Racial/Ethnic differences in inflammation levels among older adults 56+: an examination of sociodemographic differences across inflammation measure. Biodemography Soc Biol. 2024;69:75–89. 38807566 10.1080/19485565.2024.2356672 PMC11257156

[2] Ruiz M. Into the Labyrinth of the Lipocalin α1-Acid Glycoprotein. Front Physiol. 2021;12:686251. 34168570 10.3389/fphys.2021.686251 PMC8217824

[3] Wu S Teng Y Lan Y. The association between fat distribution and α1-acid glycoprotein levels among adult females in the United States. Lipids Health Dis. 2024;23:235. 39080765 10.1186/s12944-024-02223-9 PMC11290176

[4] Johnson JA Livingston TN. Differences between blacks and whites in plasma protein binding of drugs. Eur J Clin Pharmacol. 1997;51:485–8. 9112064 10.1007/s002280050235

[5] Zhou HH Adedoyin A Wilkinson GR. Differences in plasma binding of drugs between Caucasians and Chinese subjects. Clin Pharmacol Ther. 1990;48:10–7. 2369804 10.1038/clpt.1990.111

[6] Edeki T Dillon-Moore B He N. Comparison of plasma protein binding of basic drugs in black and white individuals. Am J Ther. 1996;3:611–5. 11862300 10.1097/00045391-199609000-00002

[7] Wu B Qiu L Lin Y Lin Q Pan Y. The association between the dietary inflammatory index and cardiorespiratory fitness in United States young adults: a cross-sectional study from the National Health and Nutrition Examination Study, 1999–2004. Front Nutr. 2024;11:1399640. 10.3389/fnut.2024.1442710 PMC11464452 39391678

[8] August KJ Sorkin DH. Racial/ethnic disparities in exercise and dietary behaviors of middle-aged and older adults. J Gen Intern Med. 2011;26:245–50. 20865342 10.1007/s11606-010-1514-7 PMC3043172

[9] Brown RM Tamazi S Weinberg CR Dwivedi A Mieres JH. Racial disparities in cardiovascular risk and cardiovascular care in women. Curr Cardiol Rep. 2022;24:1197–1208. 35802234 10.1007/s11886-022-01738-w

[10] Stierman B Afful J Carroll MD. National Health and Nutrition Examination Survey, 2017–March 2020 Prepandemic Data Files—Development of Files and Prevalence Estimates for Selected Health Outcomes. National Health Statistics Report No. 158. U.S. Department of Health and Human Services, Centers for Disease Control and Prevention, National Center for Health Statistics; 2023:vii + 27. 10.15620/cdc:106273 PMC11513744 39380201

[11] Khera A McGuire DK Murphy SA. Race and gender differences in C-reactive protein levels. J Am Coll Cardiol. 2005;46:464–9. 16053959 10.1016/j.jacc.2005.04.051

[12] Farmer HR Wray LA Haas SA. Race, gender, and socioeconomic variations in C-reactive protein using the health and retirement study. J Gerontol B Psychol Sci Soc Sci. 2021;76:583–95. 32064519 10.1093/geronb/gbaa027 PMC7887729

[13] Akinyemiju T Moore JX Pisu M. Mediating effects of cancer risk factors on the association between race and cancer incidence: analysis of the NIH-AARP Diet and Health Study. Ann Epidemiol. 2018;28:33–40.e2. 29217211 10.1016/j.annepidem.2017.11.003 PMC5807138

[14] Kuczmarski MF Mason MA Allegro D Zonderman AB Evans MK. Diet quality is inversely associated with C-reactive protein levels in urban, low-income African-American and white adults. J Acad Nutr Diet. 2013;113:1620–31. 24035460 10.1016/j.jand.2013.07.004 PMC3833870

[15] Lopez-Garcia E Schulze MB Fung TT. Major dietary patterns are related to plasma concentrations of markers of inflammation and endothelial dysfunction. Am J Clin Nutr. 2004;80:1029–35. 15447916 10.1093/ajcn/80.4.1029

[16] Piyathilake CJ Badiga S Chappell AR Johanning GL Jolly PE. Racial differences in dietary choices and their relationship to inflammatory potential in childbearing age women at risk for exposure to COVID-19. Nutr Res. 2021;90:1–12. 34049184 10.1016/j.nutres.2021.04.004 PMC8143979

[17] Esposito K Marfella R Ciotola M. Effect of a mediterranean-style diet on endothelial dysfunction and markers of vascular inflammation in the metabolic syndrome: a randomized trial. JAMA. 2004;292:1440–6. 15383514 10.1001/jama.292.12.1440

[18] Cavicchia PP Steck SE Hurley TG. A new dietary inflammatory index predicts interval changes in serum high-sensitivity C-Reactive protein. J Nutr. 2009;139:2365–72. 19864399 10.3945/jn.109.114025 PMC2777480

[19] Milan-Mattos JC Anibal FF Perseguini NM. Effects of natural aging and gender on pro-inflammatory markers. Braz J Med Biol Res. 2019;52:e8392. 31411315 10.1590/1414-431X20198392 PMC6694726

[20] Wyczalkowska-Tomasik A Czarkowska-Paczek B Zielenkiewicz M Paczek L. Inflammatory markers change with age, but do not fall beyond reported normal ranges. Arch Immunol Ther Exp (Warsz). 2016;64:249–54. 26283530 10.1007/s00005-015-0357-7 PMC4863028

[21] Clark DO Unroe KT Xu H Keith NR Callahan CM Tu W. Sex and race differences in the relationship between obesity and C-Reactive protein. Ethn Dis. 2016;26:197–204. 27103770 10.18865/ed.26.2.197 PMC4836900

[22] Camhi SM Stefanick ML Ridker PM Young DR. Changes in C-reactive protein from low-fat diet and/or physical activity in men and women with and without metabolic syndrome. Metabolism. 2010;59:54–61. 19709693 10.1016/j.metabol.2009.07.008 PMC2789861

[23] Fedewa MV Hathaway ED Ward-Ritacco CL. Effect of exercise training on C reactive protein: a systematic review and meta-analysis of randomised and non-randomised controlled trials. Br J Sports Med. 2017;51:670–6. 27445361 10.1136/bjsports-2016-095999

[24] Wee CC Mukamal KJ Huang A Davis RB McCarthy EP Mittleman MA. Obesity and C-reactive protein levels among white, black, and hispanic US adults. Obesity (Silver Spring). 2008;16:875–80. 18379563 10.1038/oby.2008.7 PMC2848449

[25] Singh-Manoux A Shipley MJ Bell JA Canonico M Elbaz A Kivimäki M. Association between inflammatory biomarkers and all-cause, cardiovascular and cancer-related mortality. CMAJ. 2017;189:E384–90. 27895145 10.1503/cmaj.160313 PMC5359103

[26] Adams SA Wirth MD Khan S. The association of C-reactive protein and physical activity among a church-based population of African Americans. Prev Med. 2015;77:137–40. 26007295 10.1016/j.ypmed.2015.05.010 PMC4490070

[27] Kumanyika SK. Advancing health equity efforts to reduce obesity: changing the course. Annu Rev Nutr. 20222022;42:453–80. 35417194 10.1146/annurev-nutr-092021-050805 PMC9824466

[28] Ridker PM. Inflammation, cardiovascular disease and cancer: moving toward predictive medicine. CMAJ. 2017;189:E382–3. 27895147 10.1503/cmaj.161033 PMC5359102

