# Supplementary material for: Racial disparities in systemic inflammation: The mediating role of diet and physical activity in α-1-acid glycoprotein levels in the U.S. population
Source: Medicine (Baltimore). 2025 Sep 19;104(38):e44661. doi: 10.1097/MD.0000000000044661 (PMC12459478; doi:10.1097/MD.0000000000044661)
Supplement: Supplementary file 1 [file medi-104-e44661-s001.docx]

| **Table. Multivariate analysis: Linear regressions taking the α-1-acid glycoprotein (AAG) levels as the dependent variable.** | | | | |
| --- | --- | --- | --- | --- |
|  | **Unstandardized beta** | **Standardized beta** | ***p*** | **95% Confidence Interval** |
| **Model 1: Linear regression taking the race as the independent variable (Nagelkerke R^2^ = 0.018)** | | | | |
| Race (Other hispanic vs Mexican American*) | -0.03 | -0.03 | 0.186 | -0.07 ; 0.01 |
| Race (White vs Mexican American*) | 0.02 | 0.05 | 0.119 | -0.01 ; 0.05 |
| Race (Black vs Mexican American*) | 0.01 | 0.01 | 0.667 | -0.02 ; 0.04 |
| Race (Other vs Mexican American*) | -0.07 | -0.11 | **<0.001** | -0.10 ; -0.04 |
| **Model 2: Linear regression taking the race, gender and age as the independent variable (Nagelkerke R^2^ = 0.055)** | | | | |
| Race (Other hispanic vs Mexican American*) | -0.03 | -0.03 | 0.175 | -0.07 ; 0.01 |
| Race (White vs Mexican American*) | 0.03 | 0.05 | 0.088 | -0.004 ; 0.05 |
| Race (Black vs Mexican American*) | 0.01 | 0.01 | 0.624 | -0.02; 0.04 |
| Race (Other vs Mexican American*) | -0.07 | -0.11 | **<0.001** | -0.10; -0.04 |
| Gender (Female vs male*) | 0.04 | 0.04 | **0.048** | 0.00; 0.08 |
| Age | 0.003 | 0.17 | **<0.001** | 0.002; 0.004 |
| **Model 3: Linear regression taking the race, gender, age, education, marital status and BMI as the independent variable (Nagelkerke R^2^ = 0.220)** | | | | |
| Race (Other hispanic vs Mexican American*) | -0.004 | -0.01 | 0.821 | -0.04 ; 0.03 |
| Race (White vs Mexican American*) | 0.04 | 0.07 | **0.007** | 0.01 ; 0.06 |
| Race (Black vs Mexican American*) | -0.01 | -0.02 | 0.525 | -0.04; 0.02 |
| Race (Other vs Mexican American*) | -0.02 | -0.04 | 0.143 | -0.05; 0.01 |
| Gender (Female vs male*) | -0.02 | -0.03 | 0.211 | -0.06; 0.01 |
| Age | -0.001 | -0.06 | **0.007** | -0.002; 0.00 |
| Education (9-11th grade vs less than 9th grade*) | 0.04 | 0.04 | 0.204 | -0.02; 0.10 |
| Education (high school graduate vs less than 9th grade*) | 0.04 | 0.06 | 0.170 | -0.02; 0.10 |
| Education (college graduate vs less than 9th grade*) | 0.02 | 0.04 | 0.490 | -0.04; 0.08 |
| Marital status (widowed-divorced vs married*) | 0.04 | 0.05 | **0.015** | 0.01; 0.08 |
| Marital status (never married vs married*) | -0.002 | -0.004 | 0.832 | -0.03; 0.02 |
| Body Mass Index (BMI) | 0.01 | 0.48 | **<0.001** | 0.01; 0.01 |

*Reference group. Numbers in bold indicate significant p-value.

Three regression models were estimated with AAG levels as the dependent variable.

- Model 1 included *race*.
- Model 2 included *race, gender, and age*.
- Model 3 included *race, gender, age, education, marital status, and body mass index (BMI)*.

Mexican American, male, less than 9th grade education, and married were used as reference groups. Reported are unstandardized beta coefficients, standardized beta coefficients, p-values, and 95% confidence intervals. Nagelkerke R² values are shown for each model. Bold values indicate statistical significance (p < 0.05).
